# The dynamic of asymptomatic *Plasmodium falciparum* infections following mass drug administrations with dihydroarteminisin–piperaquine plus a single low dose of primaquine in Savannakhet Province, Laos

**DOI:** 10.1186/s12936-018-2541-9

**Published:** 2018-11-03

**Authors:** Tiengkham Pongvongsa, Koukeo Phommasone, Bipin Adhikari, Gisela Henriques, Kesinee Chotivanich, Borimas Hanboonkunupakarn, Mavuto Mukaka, Pimnara Peerawaranun, Lorenz von Seidlein, Nicholas P. J. Day, Nicholas J. White, Arjen M. Dondorp, Mallika Imwong, Paul N. Newton, Pratap Singhasivanon, Mayfong Mayxay, Sasithon Pukrittayakamee

**Affiliations:** 1 Faculty of Tropical Medicine, Mahidol University, Bangkok, Thailand; 2 Savannakhet Provincial Health Department, Savannakhet, Savannakhet Province Laos; 3 Microbiology Laboratory, Lao-Oxford-Mahosot Hospital-Wellcome Trust Research Unit (LOMWRU), Vientiane, Laos; 4 Mahidol Oxford Tropical Medicine Research Unit (MORU), Faculty of Tropical Medicine, Mahidol University, Bangkok, Thailand; 5 Centre for Tropical Medicine and Global Health, Nuffield Department of Clinical Medicine, University of Oxford, Oxford, UK; 6 Institute of Research and Education Development, University of Health Sciences, Vientiane, Laos; 7 The Royal Society, Dusit Palace, Bangkok, Thailand

**Keywords:** Asymptomatic parasitaemia, *P. falciparum*, Elimination, MDA, Savannakhet, Laos

## Abstract

**Background:**

The increase in multidrug resistant *Plasmodium falciparum* infections threatens the malaria elimination goals in countries within the Greater Mekong Sub-region. A multi-pronged approach assuring access to basic malaria control measures, including insecticide-treated bed nets and early diagnosis and treatment was followed by mass drug administrations (MDA) in southern Savannakhet Province, Laos. The main objective of this study was to evaluate the effectiveness and safety of mass drug administrations as well as their effects on the dynamic of asymptomatic *P. falciparum* infections in 4 malaria endemic villages.

**Methods:**

Two villages were randomized to early MDA consisting of 3 rounds of a 3-day course of dihydroartemisinin–piperaquine with a single low dose of primaquine. In the other 2 villages MDA was deferred by 1 year. A total of 1036 residents were enrolled in early MDA villages and 883 in control villages (deferred-MDA). Tri-monthly parasitaemia surveys using uPCR were conducted for a year in the 4 villages.

**Results:**

Eighty-four percent (872/1036) of the residents participated in the MDAs, of whom 90% (781/872) completed 3 rounds of MDA (9 doses). In intervention villages, the prevalence of asymptomatic *P. falciparum* infections decreased by 85% after MDA from 4.8% (95% CI 3.4–6.4) at baseline (month 0 or M0) to 0.7% (95% CI 0.3–1.6) at month 12. In control villages there was a decrease of 33% in *P. falciparum* prevalence between M0: 17.5% (95% CI 15.9–20.3) and M12: 11.6% (95% CI 9.3–14.2). In bivariate and multivariate analyses *P. falciparum* infections were significantly reduced with early MDA (adjusted incidence rate ratios (AIRR): 0.08, CI 0.01–0.091) and completion of 3 MDA rounds (AIRR: 0.06; CI 0.01–0.66). A quarter of participants (226/872) reported adverse events of which 99% were mild.

**Conclusion:**

The study found a significant reduction in *P. falciparum* prevalence and incidence following MDA. MDA was safe, well tolerated, feasible, and achieved high population coverage and adherence. MDAs must be integrated in multi-pronged approaches such as vector control and preventive measures with a focus on specific risk groups such as mobile, migrant population and forest goers for a sustained period to eliminate the remaining parasite reservoirs.

*Trial registration* ClinicalTrials.gov Identifier: NCT01872702

## Background

The emergence and spread of resistance to artemisinins and its partner drugs in the Greater Mekong Sub-region (GMS) currently available to treat *Plasmodium falciparum,* are a threat to the control and elimination of malaria [1]. Failure to contain and eliminate multi-drug resistant malaria from the GMS could result in a public health disaster [2].

The National Malaria Control Programme of Laos relies on routine case detection and treatment in peripheral health centres [3]. Microscopy is available only in central, regional, provincial and district level health facilities and rapid diagnostic tests (RDTs) are the diagnostic mainstay [3]. Polymerase chain reaction (PCR) is not used for the routine diagnosis of malaria [4]. Artemisinin-based combination therapy (ACT) with coformulated artemether + lumefantrine (AL) was introduced as a pilot intervention for early diagnosis and treatment (EDAT) in 2005 and scaled-up gradually to cover all health facilities across the country in 2008 [5]. AL remains the first line treatment in Laos [6].

A recent study had identified artemisinin resistant *P. falciparum* strains in Phouvong District of Attapeu [1] and in two districts of Champasak Province, southern Laos [5, 6]. In response to reports of multi-drug resistant *P. falciparum* malaria, among the multi-pronged approaches for containment and elimination, Laos has been intensifying the distribution of long-lasting insecticide-treated net (LLIN) and indoor residual spraying (IRS) [7].

As a part of the national strategic plan for malaria control and elimination, Laos has adopted the goal to eliminate *P. falciparum* malaria by 2030 [4, 5, 8]. There is an urgent need to find effective interventions to rapidly reduce the *P. falciparum* malaria reservoirs from the country.

The main objective of this study was to evaluate the feasibility, safety, acceptability and impact of a pilot implementation of targeted malaria-elimination (TME) on the dynamics of asymptomatic *P. falciparum* infection in Laos. TME aims to eliminate *P. falciparum* parasites by mass drug administration (MDA) with dihydroartemisinin–piperaquine (DHA–PP) plus a single low dose of primaquine (SLPQ) in villages where basic malaria control measures (early diagnosis, appropriate treatment, and universal access to LLINs) have been established but transmission persists. The dynamic of submicroscopic *P. falciparum* parasitaemia before MDA and during the tri-monthly follow-up over a 12 months period was studied in order to provide a better understanding and objective evidence of its impact on the submicroscopic parasite reservoir. The findings from this study are expected to guide the national malaria control strategies in Laos. This article describes the impact of MDA on *P. falciparum* infections; a second report will describe the impact on *Plasmodium vivax* infections.

## Methods

### Study site and design

This was a cluster-randomized, open, controlled clinical trial conducted between April 2016 and May 2017 in Nong District in the southern Savannakhet Province of Laos. In 2014, five of the 15 districts of Savannakhet Province were classified as strata 3 (high risk) where the Annual Parasite Incidence (API) was above 10 per 1000 people at risk and Nong was the district with the second highest API (15 per 1000 people) in the province [6]. The study was part of a multicentre trial conducted in Myanmar, Vietnam, Cambodia and Laos to evaluate the impact of mass DHA/piperaquine administrations on *P. falciparum* [9].

### Community engagement and study procedures

**Fig. 1:**
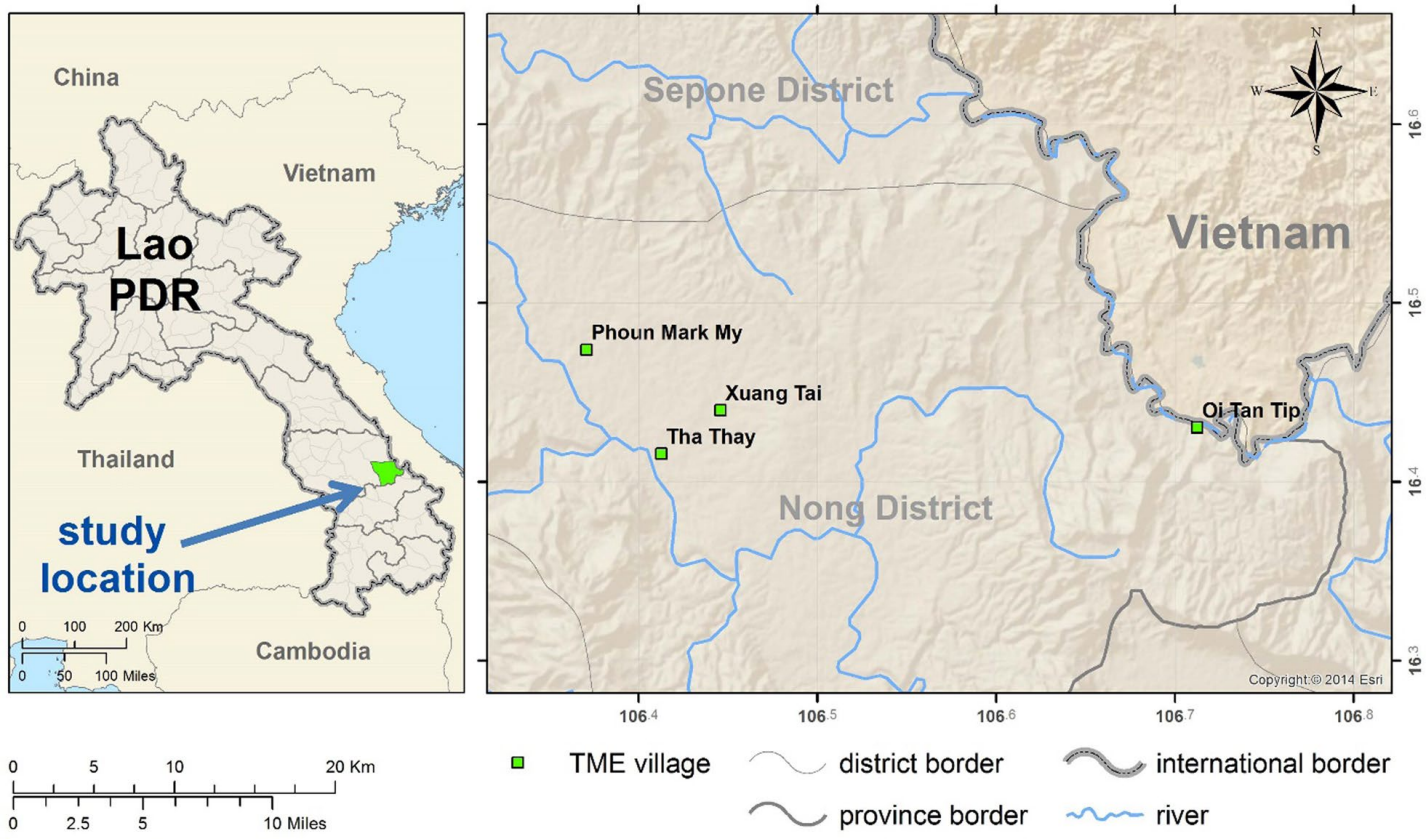
TME study sites in Savannakhet province of Laos

Initial steps in community engagement entailed comprehensive workshops with central, provincial and district level authorities to explain the purpose of the project and its procedures. These workshops were attended by representatives of the government health authorities including the Lao Womens Union, Lao Youth Organization, education and culture departments, village heads and elders [7, 10, 11]. In parallel with engagement workshops, prevalence surveys were conducted in 20 villages to determine the prevalence of malaria [4]. Based on uPCR surveys the 4 villages with the highest *P. falciparum* prevalence and enthusiasm were chosen for this study: Phoun Mak My (PMM; population: 480), Tha Thay (TT; population: 526), Xuang Tai (XT; population: 371) and Oi Tan Tip (OTP; population: 512) [4] (Fig. 1). Two villages, PMM and TT received MDA in the 1st year (intervention villages), and the remaining two villages XT and OTP received deferred MDA in the 2nd year (control villages). The intervention, early versus deferred MDA was allocated by restricted randomization within two pairs of villages matched for geographical proximity and parasite prevalence. In the control villages the coverage and the impact of the deferred MDA on *P. falciparum* infections as well as adverse events were not evaluated.

Intense community engagement activities were conducted in each village before and during the MDA as described in detail elsewhere [7, 10, 11]. All villagers who agreed to provide written informed consent (by parents or guardian in case of children) were invited to participate in the study except pregnant women, children aged less than 6 months, participants with a history of allergy or known contraindication to the study drugs and candidates who were in the opinion of the study clinician, too ill to participate. The baseline (M0) and tri-monthly follow-up surveys (M3, M6, M9 and M12) were conducted over a 1-year period.

All the eligible participants were registered and provided with a unique study number. Sociodemographic characteristics of the participants and the recent clinical history were recorded in Open Data Kit (ODK^®^) application (www.opendatakit.org) using a smartphone. All participants were provided compensation for their travel expenses and loss of income consistent with the recommendations of the local ethical committee [10].

### Mass drug administration

Following blood sampling each participant received (under direct observation) a full course of DHA–PP (7 mg/kg dihydroartemisinin and 55 mg/kg piperaquine) for 3 days plus a single low dose of PQ (0.25 mg/kg, given on day 1) on M0, M1, M2 [10]. A home visit was made by village volunteers together with TME doctors if participants developed an adverse event (AE) following the MDA [10]. All adverse events or drug-related side effects were assessed, treated and documented.

### Sample collection

During the quarterly blood surveys, a 3.0 mL blood sample was collected from participants over 5 years of age and 0.5 mL from children under 5 years [7, 10, 11]. The blood samples were collected in EDTA anticoagulated tubes and stored in ice packed cool boxes and transported to a centralized field laboratory in the district within 12 h of blood collection. Upon return to the centralized laboratory, whole blood was separated and the red blood cell pellets were promptly frozen and stored at − 30 °C for up to 7 days. Each sample was labelled with a barcode and negative controls were added to the sample pool. The samples were transported on dry ice to the molecular laboratory of the Mahidol Oxford Tropical Medicine Research Unit (MORU) in Bangkok, Thailand for uPCR analysis. All participants were diagnosed on sites for malaria using the SD Bioline Ag *P. falciparum*/Pan (Standard Diagnostics Inc.) rapid diagnostic test (RDT). Those with positive RDTs were treated with artemether/lumefantrine, as per the Lao national treatment guidelines. RDT tests were performed and interpreted by an experienced laboratory technician following the manufacturer’s recommendation.

### DNA extraction and PCR amplification

DNA was extracted from thawed packed red blood cells using an automated DNA extraction machine (QIAsymphony and DPS DNA midi kit; Qiagen, Germany). The DNA was dried, concentrated and then used as a template for PCR detection and quantification of *Plasmodium*. Quantitative PCR (uPCR) analysis was performed as described elsewhere [12]. Briefly, DNA of *Plasmodium* was detected and quantified using 18S rRNA-targeting primers. For *Plasmodium* positive samples, an attempt was made to identify the species using *P. falciparum* and *P. vivax* specific PCR primers as described [12].

### Statistical analysis

The socio-demographic characteristics of the participants was described using frequency and percentage for the categorical variables while median and interquartile range (IQR) were used for the continuous variables except for parasite density which was analysed by using geometric mean and 95% confidence interval (CI). The Chi squared test was used to test the statistical difference of normally distributed groups and the Wilcoxon rank-sum test to analyse was used to test the statistical difference of the median or distribution between groups with skewed distribution.

The prevalence and 95% confidence intervals of *P. falciparum* infection for each 3-month interval was analysed using the number of people with *P. falciparum* or mixed infections as numerator and number of villagers for whom a uPCR results were available as denominator. The prevalence of *P. falciparum* infection at each time point after month 3 and over time was compared between early MDA and deferred-MDA villages using multilevel logistic regression models clustered by villages and adjusted for repeated observations.

The incidence rate (person-years) and 95% confidence intervals of *P. falciparum* infection for each 3-monthly period was estimated using the number of people found infected divided by their exposure time (person-year). The incidence rate for each time point after month 3 and over time was compared between early MDA and deferred-MDA villages using multilevel Poisson regression models clustered by villages and adjusted repeated observations.

The MDA coverage was estimated as the proportions of villagers who completed the MDA at month 0, 1, and 2 and for all 3 MDA rounds. Participation was categorized as “not received MDA”, “not completed MDA”, “completed 3 days MDA”, “taken any dose in 3 rounds”, and “completed all 3 rounds” using all villagers living at the time of the MDA in villages as denominator. The blood sampling coverage was estimated as number of people who gave blood sample in each 3-monthly survey with number of residents at the time of the survey in the early MDA and deferred-MDA villages.

A univariate analysis was performed to obtain the unadjusted estimates of incidence rate ratios (IRR) of infections and MDA status as well as associations with each of the baseline variables including sex, age, fever, bed net use and season. A multivariable analysis was used to obtain the adjusted estimates. The impact of the MDA was examined using a multilevel mixed effect Poisson model to obtain the IRR of *P. falciparum* infection with 95% confidence interval. Multilevel mixed effect modelling allowed adjusting for village, villager and repeated measurements specific random effects. In the multilevel model, level 1 was repeated measurements of villagers over time, level 2 was villagers, and level 3 was village. To assess the impact of the treatment and prophylactic effect of the anti-malarial drugs a secondary analysis was conducted in which the first 3 months of surveillance in the intervention villages was omitted.

A p-value < 0.05 was considered statistically significant. All analyses were performed using Stata, version 14 (StataCorp LLC, College Station, Texas). The seasonal variation in *P. falciparum* infections at each time point of both MDA and non-MDA villages was described using monthly rainfall data of Nong district provided by the Savannakhet Meteorological office.

### Ethical consideration

The study protocol was approved by the National Ethic Committee for Health Research (013NIOPH/NECHR. 15th February, 2016), Ministry of Health, Laos, the Oxford Tropical Research Ethics Committee (OXTREC-1017-13. 9th April, 2015), and Ethical Committee of the Faculty of Tropical Medicine, Mahidol University (TMCD 00754. 8th Dec, 2016).

## Results

### Baseline characteristics

**Fig. 2:**
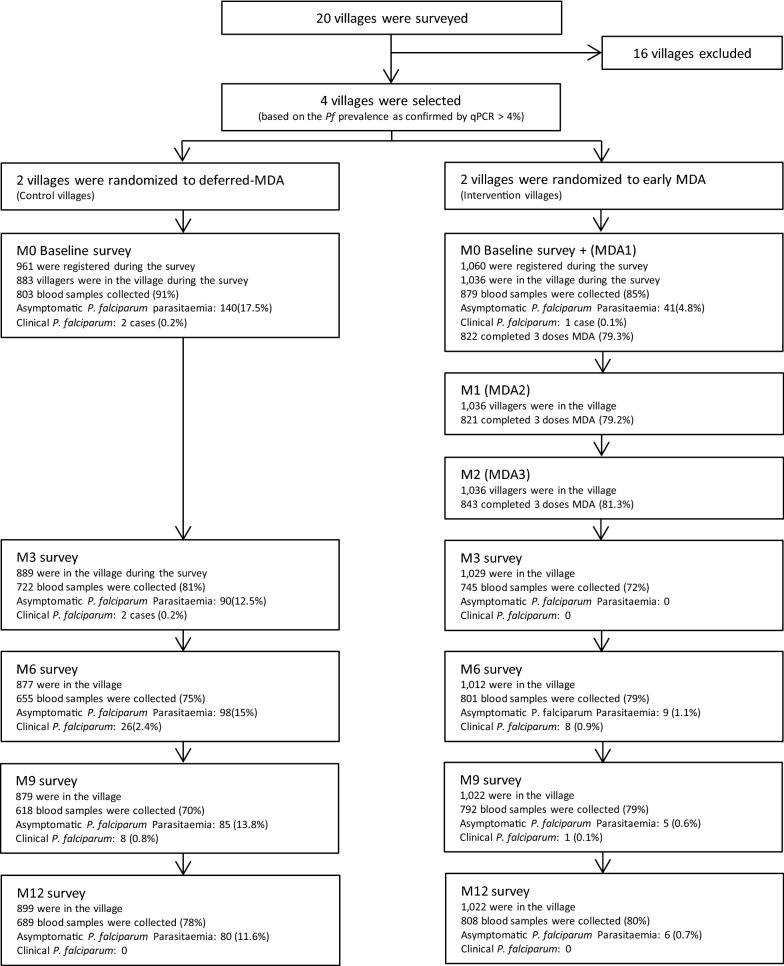
Study overview

A total of 2021 participants were registered and participated in the baseline survey in four selected villages, of which 52% (1060/2021) were from intervention (early MDA) villages and 48% (961/2021) from control (deferred MDA) villages (Fig. 2). The majority of participants in both early MDA (80%; 871/1036) and deferred-MDA villages (84%; 810/883) (p = 0.046) reported that they had worked in the forest during the last 3 months. More than half of the population used a bed net in both early MDA (56%; 498/887) and deferred-MDA villages (62%; 506/811; p = 0.030) villages (Table 1).

**Table 1:** Baseline characteristics of the study participants

Characteristics	Early MDA-intervention villages	Deferred-MDA control villages	p-value^a^
	(N = 1060)	(N = 961)	
Gender, n (%)			0.981
Male	537 (51)	462 (51)	
Female	523 (49)	499 (49)	
Age, median (IQR)	17 (8–32)	15 (6–30)	< 0.001
Age group (years), n (%)			0.644
< 10	345 (33)	319 (35)	
10–19	241 (23)	204 (22)	
20–39	289 (27)	243 (27)	
40 or more	185 (17)	145 (16)	
Occupation, n (%)			0.005
Farmer	579 (55)	461 (51)	
Child	198 (19)	231 (25)	
Student	231 (22)	181 (20)	
Others	52 (5)	38 (4)	
Type of resident, n (%)			0.783
Permanent	1042 (98)	898 (99)	
Temporary	18 (2)	13 (1)	
Villagers in village during the survey, n (%)	1036 (98)	883 (97)	
Stay overnight in forest, n (%)	(n = 871)	(n = 810)	0.046
No	766 (88)	732 (90)	
Yes, < 2 weeks ago	78 (9)	67 (8)	
Yes, ≥ 2 weeks	27 (3)	11 (1)	
Weight (kg) median (IQR)	(n = 888)	(n = 811)	0.075
	40 (19–49)	38 (18–46)	
Height (cm), median (IQR)	(n = 886)	(n = 811)	< 0.001
	144 (118–153)	143 (115–152)	
Temperature (c), median (IQR)	(n = 888)	(n = 811)	< 0.001
	36.9 (36.7–37.1)	36.9 (36.7–37.0)	
Fever on registration date, (T ≥ 37.5 °C)	43 (5)	27 (3)	0.117
Fever^b^	120 (14)	146 (18)	0.011
Bed net use, n (%)	(n = 887)	(n = 811)	0.030
Regular	498 (56)	506 (62)	
Irregular	322 (36)	249 (31)	
Never use	67 (8)	56 (7)	
Asymptomatic Pf prevalence, n (%)	(n = 859)	(n = 802)	< 0.001
	41 (4.8)	140 (17.4)	
Pf clinical cases, n (%)	1 (0.1%)	2 (0.2%)	
Pf density^c^, geometric mean (95% CI)	10,864 (1676–20,795)	61,426 (42,542–88,693)	< 0.001

Median (IQR) tested using Wilcoxon rank-sum test

^a^N (%) tested using Chi square test or Fisher’s exact test (f)

^b^History of fever combined with Temp ≥ 37.5

^c^Test in log scale using Student’s test

### Compliance with blood sampling, follow-up, and MDA coverage

The overall percentage of the villagers who participated in blood sampling at least once was 88% (948/1073) in early MDA villages and 93% (875/936) in deferred-MDA (p < 0.001). Among the 1073 villagers, 36 (3.3%) were not eligible for the mass drug administration. The percentage of the participants who took at least one dose of MDA was 84% (872/1036) and 90% (781/872) took the full nine doses in all 3 rounds in the early MDA villages. The percentage of the participants who had completed 3 days was 79.3% (822/1036) at M0, 79.2% (821/1036) at M1 and 81.3% (843/1036) at M3. The percentage of the participants who had not completed any MDA was 2% (20/1036) at M0, 1% (10/1036) at M1 and 0.6% (6/1036) at M2 (Table 2).

**Table 2:** Compliance of blood sampling, follow-up and MDA coverages

Characteristics	Baseline	Follow-up time						
	M0 (MDA1)	M1 (MDA2)	M2 (MDA3)	M3	M6	M9	M12	Overall^b^
Number of villagers in village during the survey^a^								
Intervention village (early MDA)	1036	1036	1036	1029	1012	1022	1022	1073
Control village (deferred MDA)	883			889	877	879	899	936
Blood sampling coverage, n (%)								
Intervention village (early MDA)	879 (85)			745 (72%)	801 (79%)	792 (77%)	808 (79%)	948 (88%)
Control village (deferred MDA)	803 (91)			722 (81%)	655 (75%)	618 (70%)	689 (77%)	875 (93%)
p-value								< 0.001
MDA coverage in intervention village, n (%)								
Not received MDA	194 (19.0)	205 (19.8)	187 (18.0)					
Not completed MDA	20 (2.0)	10 (1.0)	6 (0.6)					
Completed 3 days MDA	822 (79.3)	821 (79.2)	843 (81.3)					
People who took any dose in 3 rounds, n (%)			872 (84)					
Completed 3 rounds (9 doses)			781 (90)					

^a^Excluded people away

^b^At least participated once

### The effects of MDA on the prevalence and incidence of asymptomatic *P. falciparum* in the early MDA villages as confirmed by uPCR

**Fig. 3:**
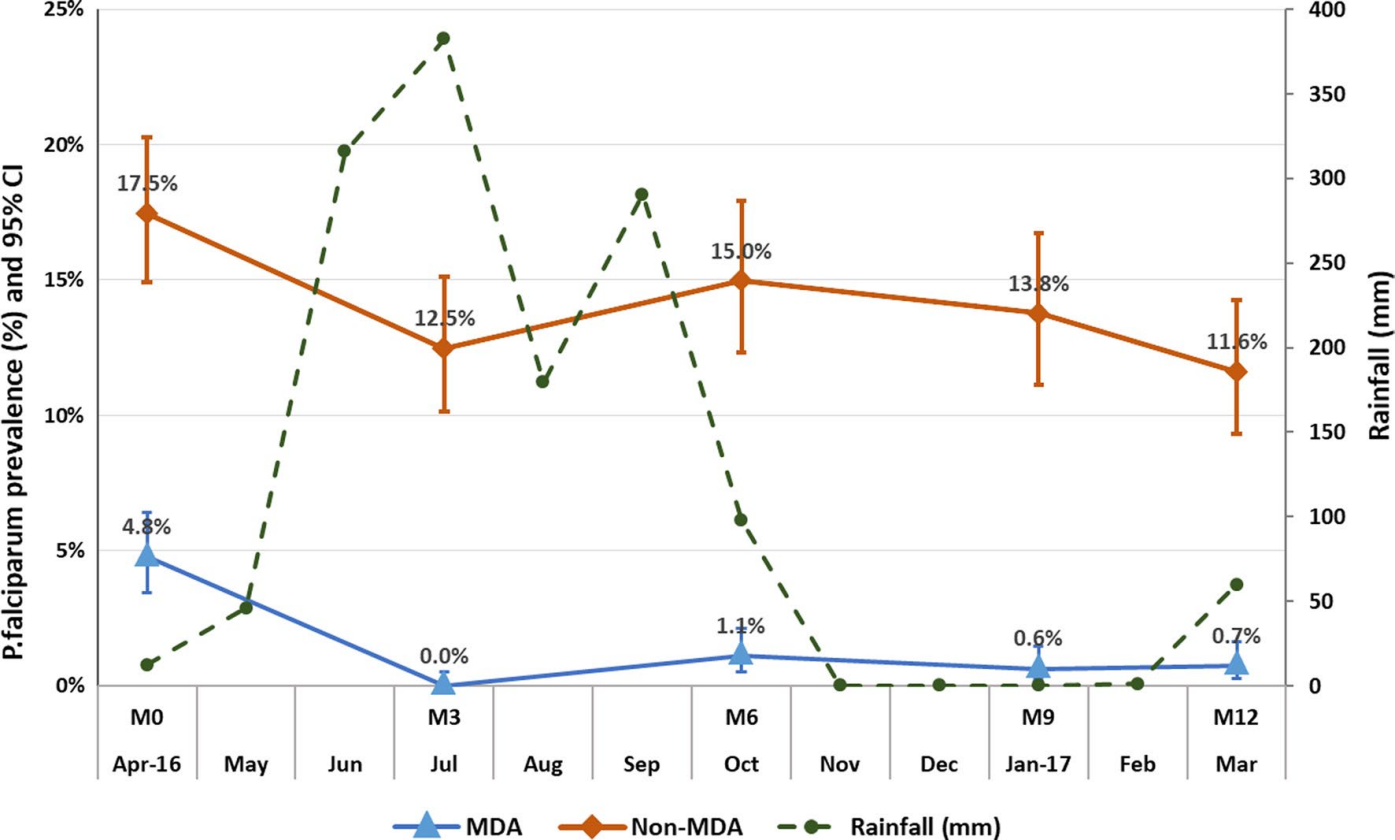
Seasonal variation of asymptomatic *P. falciparum* parasitaemia prevalence during tri-monthly follow-up survey in MDA and non-MDA villages

In early MDA villages, the prevalence (95% CI) of asymptomatic *P. falciparum* parasitaemia at baseline (M0) was 4.8% (3.4–6.4; Fig. 3). The prevalence of *P. falciparum* infections during tri-monthly follow-up was 0% (0–0.5) at M3, 1.1% (0.5–2.1) at M6, 0.6% (0.2–1.5) at M9 and 0.7% (0.3–1.6) at M12. In deferred-MDA villages, *P. falciparum* prevalence was 17.5% (15.9–20.3) at baseline. The prevalence of *P. falciparum* parasitaemia was declined significantly over time in early MDA villages (AOR = 0.82; CI 0.74–0.86, p < 0.001) but this was not significant in deferred-MDA villages (AOR: 0.97; CI 0.94–1.01, p = 0.101) (Table 3).

**Table 3:** Prevalence and incidence of asymptomatic *P. falciparum* parasitaemia at baseline and during tri-monthly follow-up

Variables	Village status	Baseline	Follow-up time				Over follow-up time
		M0	M3	M6	M9	M12	
Number of villagers*	Early MDA	859	745	801	792	808	
	Deferred-MDA	802	722	655	618	689	
Number of Pf infections	Early MDA	41	0	9	5	6	
	Deferred-MDA	140	90	98	85	80	
Pf prevalence, % and (95% CI)	Early MDA*	4.8 (3.4–6.4)	0 (0–0.5)	1.1 (0.5–2.1)	0.6 (0.2–1.5)	0.7 (0.3–1.6)	
	Deferred-MDA	17.5 (15.9–20)	12.5 (10.1–15.1)	15 (12.3–17.9)	13.8 (11.1–16.7)	11.6 (9.3–14.2)	
Comparison between group, AOR (95% CI, p-value) **				0.18 (0.01–3.38, 0.254)	0.07 (0.01–1.26, 0.072)	0.13 (0.01–2.37, 0.170)	0.42 (0.01–12.28, 0.611)
Pf exposure time (person-years)	Early MDA	N/A	183	196	192	198	
	Deferred-MDA	N/A	176	159	151	167	
Incidence of Pf infection (per 1000 person-years)	Early MDA	N/A	0 (0–20)	46 (21–87)	26 (8–61)	30 (11–66)	
	Deferred-MDA	N/A	510 (410–627)	617 (501–752)	563 (450–696)	478 (379–595)	
Comparison between group, AIRR (95% CI, p-value)**				0.21 (0.01–3.08, 0.254)	0.09 (0.01–1.24, 0.071)	0.15 (0.01–2.24, 0.169)	0.08 (0.01–0.88, 0.039)

*Significant declining over time in early MDA group, (AOR = 0.82, 95% CI 0.74–0.86, p-value < 0.001) but this was not significant in Non-MDA group, (AOR = 0.97, 95% CI 0.94–1.01, p-value = 0.101)

**Adjusted for Pf prevalence at baseline (village level) and cluster (village) effect

**Fig. 4:**
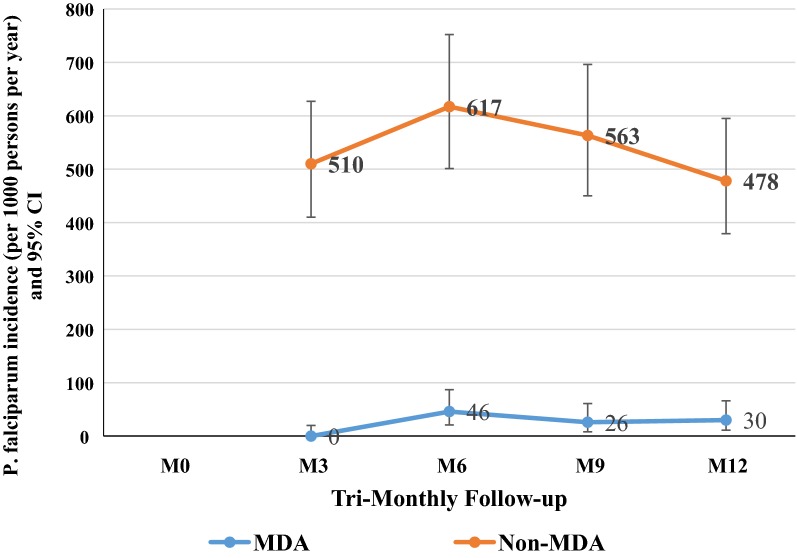
Comparison of asymptomatic *P. falciparum* parasitaemia incidence per 1000 person-years between intervention villages (MDA) and control villages (non-MDA) at tri-monthly basis during a period of 1-year. The orange line is the tri-monthly mean of asymptomatic *P. falciparum* parasitaemia incidence (per 1000 person-years) 95% CI in non-MDA villages. The blue line is the tri-monthly mean of asymptomatic *P. falciparum* parasitaemia incidence 95% CI in MDA villages

The incidence rate (95% CI) of *P. falciparum* infections per 1000 person-years in early MDA village was 0/1000 (0**–**20) at M3, increased to 46/1000 (21**–**87) at M6, 26/1000 (8**–**61) at M9 and remained 30 (11**–**66) at M12 (Fig. 4). The incidence rate in the deferred villages was 510/1000 (410**–**627) at M3, 617/1000 (501**–**752) at M6, 563/1000 (450**–**696) at M9 and 478/1000 (379**–**595) at M12 (AIRR: 0.08; CI 0.01**–**0.88, p = 0.039; Table 3 and Fig. 4).

The incidence rate was significantly lower in people who had completed 3 MDA rounds than in people who had completed < 3 rounds of MDA (AIRR: 0.06, 95% CI 0.01–1.70; p = 0.022) **(**Table 4). If the infections that occurred during the first 3 months of surveillance in the intervention villages is omitted the AIRR is 0.11 (95% CI 0.01–1.16).

**Table 4:** Multilevel mixed-effects Poisson regression on *P. falciparum* infections in early MDA village and compared with deferred-MDA village during tri-monthly follow-up after MDA

Characteristics	IRR (95% CI)	p-value	Model 1: MDA intervention Adjusted IRR (95% CI)^a^	p-value	Model 2: MDA coverage Adjusted IRR (95% CI)^a^	p-value
Intervention						
MDA	0.08 (0.01–0.88)	0.039	0.08 (0.01–0.91	0.042		
Non-MDA	Reference		Reference			
Coverage						
MDA completed 3 rounds	0.06 (0.01–0.64)	0.020			0.06 (0.01–0.066)	0.022
MDA completed 1 and 2 rounds	0.11 (0.01–1.74)	0.118			0.11 (0.01–1.70)	0.114
MDA not completed in a single round/No MDA	0.50 (0.04–6.27)	0.591			0.52 (0.04–6.47)	0.611
Non-MDA	Reference				Reference	
Gender						
Male	1.00 (0.72–1.39)	0.990	1.01 (0.73–1.39)	0.963	1.03 (0.74–1.41)	0.878
Female	Reference		Reference		Reference	
Age in year	0.99 (0.98–1.00)	0.088	0.99 (0.98–1.00)	0.087	0.099 (0.98–1.00)	0.089
Fever	0.91 (0.50–1.67)	0.759				
Bed net use						
Regular	Reference					
Irregular	1.07 (0.80–1.43)	0.646				
Never use	1.10 (0.58–2.07)	0.775				
Season						
Wet	1.04 (0.84–1.28)	0.725	1.04 (0.84–1.28)	0.724	1.05 (0.85–1.29)	0.667
Dry	Reference		Reference		Reference	

*IRR* incidence rate ratio

^a^Adjusted for gender, age, fever and season

### The effects of MDA on the prevalence and incidence of clinical *P. falciparum* malaria as detected by RDT

Over the 12 months surveillance period 10 falciparum malaria cases, defined as fever and confirmed infection were diagnosed in early MDA villages and 38 in villages with deferred MDA (Table 5). The prevalence (95% CI) of falciparum malaria cases was 0.0% (0.0–0.4) at M3, 0.9% (0.4–1.7) at M6, 0.1% (0.0–0.6) at M9 and zero percent at M12 in the early MDA village. In the deferred-MDA village, the falciparum malaria prevalence was 0.2% (0.2–0.7) at M3, 2.4% (6–3.5) at M6, 0.8% (0.3–1.5) at M9 and zero percent at M12. The difference in falciparum malaria prevalence between early MDA and deferred-MDA villages was statistically significant at M6 and M9 (p = 0.008 and p = 0.035, respectively). The incidence per 1000 person-years of clinical *P. falciparum* malaria at M6 and M9 was significantly lower in the early MDA than in the deferred -MDA villages (p = 0.011 and p = 0.037, respectively) (Table 5).

**Table 5:** Prevalence and incidence of symptomatic *P. falciparum* in early MDA and deferred-MDA villages during tri-monthly follow-up

Variables	Village status	Baseline	Follow-up time			
		M0	M3	M6	M9	M12
Number of villagers^a^	Early MDA	883	889	877	879	899
	Deferred-MDA	1036	1029	1071	1022	1022
Number of Pf clinical case^b^	Early MDA	1	0	8	1	0
	Deferred-MDA	2	2	26	8	0
Prevalence of Pf clinical case 95% CI	Early MDA		0.0 (0.0–0.4)	0.9 (0.4–1.7)	0.1 (0.0–0.6)	0
	Deferred-MDA		0.2 (0.2–0.7)	2.4 (1.6–3.5)	0.8 (0.3–1.5)	0
p-value			0.192	0.008	0.035	
PF exposure time (person-year)	Early MDA	N/A	222	219	220	220
	Deferred-MDA	N/A	257	268	256	205
Incidence of clinical Pf infection 95% CI (per 1000 person-years)	Early MDA	N/A	0 (0–6.6)	36 (16–72)	4.6 (0.1–25.3)	0
	Deferred-MDA	N/A	8 (0.9–28.1)	97 (63–142)	31 (13.5–61.6)	0
p-value			0.288	0.011	0.037	

^a^Number of villagers who have RDT result

^b^Confirmed by RDT

### Seasonal variability of the prevalence of asymptomatic *P. falciparum* parasitaemia

The prevalence of asymptomatic *P. falciparum* parasitaemia increased in April (M0) (17.5% in deferred MDA and 4–8% in early MDA villages) and in October (M6) (15% in deferred MDA and 1.1% in early MDA villages). The falciparum prevalence increased in deferred MDA villages following the peak rainfall in July 2016 and slightly decreased during the dry season from November to February (Fig. 3).

### Recurrent and persistent *P. falciparum* infections

The proportion of participants who had *P. falciparum* infections at M0 and were cured during follow-up at M3, M6, M9 and M12 was higher in early MDA village (40/41; 98%) than in deferred MDA village (39/140; 28%; p < 0.001). In deferred MDA villages 26/140 (18.5%) participants were found to be infected with *P. falciparum* once, 32/140 (22.8%) twice, 22/140 (15.7%) three times and 21/140 (15%) four times. In the early MDA villages only one (2.5%) participant was infected with *P. falciparum* three times during tri-monthly follow-up (Table 6).

**Table 6:** Recurrent and persistent of *P. falciparum* infections during tri-monthly follow-up (M3, M6, M9 and M12) in early and deferred MDA villages

Village status	Number of *P. falciparum* infections at M0	Number of *P. falciparum* infections during all follow-up time points (M3, M6, M9 and M12)				
		Uninfected	Infection 1 time	Infection 2 times	Infection 3 times	Infection 4 times
Deferred MDA village, n (%)	140	39 (27.8)	26 (18.5)	32 (22.8)	22 (15.7)	21 (15.0)
Early MDA village, n (%)	41	40 (97.5)	0	0	1 (2.5)	0
p-value^a^		p < 0.001				
Total	181	79 (43.6)	26 (32.0)	32 (17.6)	23 (12.7)	21 (11.6)

^a^Using z test or Chi square test

### Adverse events

Adverse events were treated and followed-up in early MDA villages. 282 of 872 (32%) reported 295 adverse events following participation in the MDAs. The most frequent complaints were common cold (17.3%; 51/295), gastritis (7.5%; 22/295), diarrhoea (7.5%; 22/295), vomiting (6.8%; 20/295), dizziness (6.1%; 18/295), pruritus (6.1%; 18/295), watery stool (4.1%; 12/295), nausea (2.7%; 8/295), headache (2.7%; 8/295) and others (38%; 112/295). Most adverse events (98.6%; 291/295) were mild, 3 (1.0%) adverse events were moderate and 1 (0.3%) adverse event was severe, a case of pneumonia was considered severe and required hospitalization (Table 7).

**Table 7:** Type of adverse events following mass administrations of dihydroartemisinin-piperaquine plus single low dose of primaquine

Type of adverse events	Number of people with events	Number of events	% of event
	n = 282	n = 295	
Common cold	50	51	17.3
Gastritis	22	22	7.5
Diarrhea	22	22	7.5
Vomiting	20	20	6.8
Dizziness	18	18	6.1
Pruritus	16	18	6.1
Watery stool	12	12	4.1
Nausea	8	8	2.7
Headache	8	8	2.7
Cough	4	4	1.4
Others	102	112	38.0

## Discussion

The emergence of artemisinin and partner drug resistance leaves few treatment options for countries in the Greater Mekong Sub-region, including Laos [1, 2, 13, 14]. This is a first study in Laos evaluating the impact, feasibility, safety and effectiveness of a MDA consisting of DHA–PP and single low dose primaquine as a potential tool to eliminate *P. falciparum* parasitaemia.

For a MDA to be successful, a high population coverage is essential and mathematical modellers have suggested that at least 80% population coverage is required for MDA to interrupt the local malaria transmission [15–17]. In early MDA villages alone 84% (872/1036) of the residents participated in the MDA and of those who participated 90% (781/872) completed all 3 rounds (9 doses). Achieving such a high coverage required concerted action of all involved including intensive community engagement, provision of ancillary care, monetary and non-monetary incentives and the factors embedded in local social and cultural context such as cohesive nature of the communities and decision making dynamics within the households [7, 10, 11].

*Plasmodium* infections were actively monitored every 3 months for a year in both intervention villages and control villages. Consistent with observations in Myanmar and Cambodia the incidence and prevalence of *P. falciparum* infections was suppressed in villages after the early MDAs [13, 18]. In Laos the effect of MDAs persisted throughout the follow up period.

Future roll out of MDA in Laos may be able to interrupt the transmission of *P. falciparum* if high coverage can be assured and basic malaria control measures such as access to LLIN and early diagnosis and treatment is assured. A recent MDA in Zambia employed only 2 rounds of DHA–PP and showed a substantial albeit temporary impact on malaria prevalence, cumulative infection incidence and confirmed case incidence rates over a 5 months follow-up period [19]. A scale-up of targeted malaria elimination which includes the basic malaria control measures combined with MDA in hotspots has recently been successfully implemented in Thai-Myanmar border areas [20]. MDA in the real world of malaria control programmes may become more feasible and acceptable as neither blood sampling nor follow up are required.

Two systematic reviews of MDAs suggest that MDA interrupts the malaria transmission temporarily followed by a rebound in malaria prevalence (but not to the level of pre-intervention). In isolated geographical locations such as islands, where introduction of new malaria cases are low due to minimal or no migration the impact of MDAs is longer lasting [16, 17]. So far, only one study in Vanuatu Island has shown permanent interruption of malaria transmission following MDAs [21, 22].

In general, MDA was well tolerated and safe. Sub-studies accompanying this study reported that adverse events were conflated in villagers’ perception with pre-existing illnesses. Community engagement that accompanied this study provided free health care in these villages which could mean that villagers felt more encouraged to attend the health centres and the services provided owing to the fact that economical barriers were removed [7, 10, 11]. In a scale up programme in Thai-Myanmar border areas, which includes MDAs, monitoring the adverse events of anti-malarials was critical in the successful implementation of malaria posts and MDAs [20]. Future studies could benefit from exploring factors related to the perception of adverse events and ways to mitigate the negative effect of adverse events on the perception of MDAs. In the current study, adverse events triggered deployment of additional doctors (who stayed in the villages until the study finished) and health centre staff who were mobilized to provide health care [7, 10, 11].

### Strengths and limitations

This study randomised only 4 villages into 2 arms with 2 villages in each arm. Despite the limited statistical power the current study was able to show meaningful differences between study arms. A large multi-centre study of which the current study was part of, has just been completed in the GMS and will provide more robust evidence for the effectiveness of the intervention. The current study could have benefitted by incorporating detailed entomological observations to test the impact in terms of the human biting rate, sporozoite index, and entomological inoculation rate of MDA on vectors [20]. Future implementation of MDA in malaria control programmes will require detailed cost efficacy analyses to guide policy makers. In the current pilot study, the implementation team and the government authorities invested considerable resources including intensive community engagement to achieve high coverage, not only in the participation in MDAs but also in the intensive monitoring and evaluation of the intervention. In scaling up of MDAs, intense community engagement with allocation of incentives for participation may neither be possible, nor required but could ultimately affect participation. The current project was initiated by researchers and relied on the enthusiasm of the villagers. Different forces, e.g. conformity may play a more important role once the programme is part of a national malaria elimination programme. Scaling up the programme without frequent blood collections, may also increase the community participation.

## Conclusions

In a remote, malaria endemic region of Savannakhet Province, Laos, MDA consisting of 3 rounds DHA–piperaquine with a single low dose of primaquine at monthly intervals was found safe, well tolerated and feasible when accompanied by intensive community engagement. Following the intervention MDA, sub-microscopic malaria prevalence was significantly reduced in intervention villages compared to control villages. A high population coverage with adherence to all 3 rounds was critical for this success. Achieving high population coverage in remote communities requires accompanying community engagement. For future roll out, an adequate community engagement strategy entailing community collaboration and sharing of responsibility with community members is critical. This study shows significant reduction in *P. falciparum* prevalence and incidence following MDA. MDA can become an effective intervention tool for malaria elimination in Savannakhet Province and potentially other parts of Laos and the GMS. Interruption of transmission and thus malaria elimination can only be achieved if the re-importation of malaria can be prevented. Future studies should integrate MDAs with multi-pronged approaches such as vector control (e.g. ivermectin) and preventive measures (e.g. anti-malarial vaccines) with a focus on high risk groups, such as mobile populations and forest goers.
